# The Coexistence of Hypertension and Ovariectomy Additively Increases Cardiac Apoptosis

**DOI:** 10.3390/ijms17122036

**Published:** 2016-12-06

**Authors:** Yi-Yuan Lin, Yu-Jung Cheng, Jun Hu, Li-Xi Chu, Woei-Cherng Shyu, Chung-Lan Kao, Tzer-Bin Lin, Chia-Hua Kuo, Ai-Lun Yang, Shin-Da Lee

**Affiliations:** 1 Graduate Institute of Clinical Medical Science, China Medical University, Taichung 40402, Taiwan; charlet8116@gmail.com; 2 Department of Physical Therapy, Graduate Institute of Rehabilitation Science, China Medical University, Taichung 40402, Taiwan; s5888116@gmail.com (Y.-J.C.); kuochiahua@gmail.com (C.-H.K.); 3 School of Rehabilitation Science, Shanghai University of TCM, Shanghai 201203, China; jasonhwu@126.com (J.H.); chulixi2@126.com (L.-X.C.); 4 Translational Medicine Research Center, China Medical University Hospital, Taichung 40402, Taiwan; shyu9423@gmail.com; 5 Graduate Institute of Immunology, China Medical University, Taichung 40402, Taiwan; 6 Center for Neuropsychiatry, Department of Neurology, China Medical University Hospital, Taichung 40402, Taiwan; 7 Department of Physical Medicine and Rehabilitation, Taipei Veterans General Hospital, Taipei 11217, Taiwan; clkao@vghtpe.gov.tw; 8 Department of Physical Medicine and Rehabilitation, School of Medicine, National Yang-Ming University, Taipei 11221, Taiwan; 9 Department of Physiology, School of Medicine, College of Medicine, Taipei Medical University, Taipei 11031, Taiwan; tblin2@tmu.edu.tw; 10 Graduate Institute of Basic Medical Science, College of Medicine, China Medical University, Taichung 40402, Taiwan; 11 Department of Biotechnology, Asia University, Taichung 41354, Taiwan; 12 Department of Sports Sciences, University of Taipei, Taipei 11153, Taiwan; yangailun@gmail.com; 13 Department of Occupational Therapy, Asia University, Taichung 41354, Taiwan

**Keywords:** menopausal hypertension, heart, caspase, cell death

## Abstract

To investigate whether the coexistence of hypertension and ovariectomy will increase cardiac Fas receptor and mitochondrial-dependent apoptotic pathways, histopathological analysis, the TUNEL assay and Western blotting were performed on the excised hearts from three groups of female spontaneously hypertensive rats (SHR), which were divided into a sham-operated group (SHR-Sham), bilaterally ovariectomized group (SHR-OVX) and normotensive Wistar Kyoto rats (WKY). Compared with the WKY group, the SHR-Sham group exhibited decreased protein levels of ERα, ERβ, p-Akt/Akt, Bcl-2, Bcl-xL and p-Bad and decreased further in the SHR-OVX group, as well as protein levels of t-Bid, Bak, Bad, Bax, cytochrome *c*, activated caspase-9 and activated caspase-3 (mitochondria-dependent apoptosis) increased in the SHR-Sham group and increased further in the SHR-OVX group. Compared with the WKY group, protein levels of Fas ligand, TNF-α, Fas death receptors, TNFR1, FADD and activated caspase-8 (Fas receptor-dependent apoptosis) increased in the SHR-Sham group, but did not increase in the SHR-OVX group, except Fas ligand and TNF-α. The coexistence of hypertension and ovariectomy attenuated the estrogen receptor survival pathway and appeared to additively increase the cardiac mitochondria-dependent, but not the Fas receptor-dependent apoptosis pathway, which might provide one possible mechanism for the development of cardiac abnormalities in hypertensive postmenopausal women.

## 1. Introduction

A four-fold increase in the incidence of hypertension occurs in postmenopausal women compared to in premenopausal women [1]. Especially, this question is particularly important when hypertensive women reach the postmenopause period. Studies show that menopause-related estrogen-deficiency causes left ventricular hypertrophy, dilated cardiomyopathy and systolic dysfunction, which have been linked to the rapid increase in the risk of developing heart failure [2,3].

Estrogen is known to have multiple protective effects in cardiovascular function [4,5]. Previous studies indicate that estrogen-deficiency or aging-related defects are associated with adverse cardiac remodeling [6,7], but the mechanisms involved remain unclear. Cardiomyocytes and cardiac fibroblasts contain estrogen receptor isoforms (ERα and ERβ) [8]. Estrogen acts through binding to estrogen receptors ERα and ERβ, which are able to mediate the stimulation of the phosphatidylinositol 3-kinase (PI3K) and protein kinase B (Akt) pathway [4,9]. Moreover, the pro-survival protein Akt also activates the Bcl-2-related anti-apoptotic pathway, Bcl-2, B-cell lymphoma-extra-large (Bcl-xL) and phosphorylated-Bad (p-Bad) and prevents the apoptosis repressor [10].

Apoptosis is triggered by the two major pathways of the intrinsic mitochondria-dependent and extrinsic Fas receptor-dependent apoptotic pathways [10,11]. The mitochondria are the main sites of action for members of the apoptosis-regulating protein truncates Bid (t-Bid), which translocate to induce the oligomerization of Bcl-2 antagonist or killer (Bak), Bcl-2-associated death promotor (Bad) and Bcl-2-associated X protein (Bax). These pro-apoptotic proteins can enhance cytochrome *c* release from the mitochondria into the cytosol, which is responsible for activating caspases -9 and -3, expediting the apoptotic progression [10,12,13]. Fas ligand or TNF-α binding with its death receptors on the cell membrane and the formation of a death-inducing signaling complex through recruitment of Fas-associated death domain (FADD), resulting in caspase 8 activation, leads to caspase-3 cleavage, which executes the cell death program [10]. Several studies have shown that deficiency of estrogen promotes cardiac apoptosis-related death, which may worsen cardiac dysfunction and heart failure [14,15,16]. Moreover, cardiomyocyte apoptosis is a very critical early pathological feature of chronic disease and the development of heart failure [17,18,19,20]. Cardiomyocyte apoptosis is increased in cardiac diseases or heart failure and is regarded as a marker of poor cardiovascular outcomes [17,21]. However, the mechanism of cardiac apoptosis in the coexistence of hypertension and ovariectomy is not understood.

Previous studies have suggested that cardiomyocytes apoptosis is mediated by Bcl-2 and Bax genes and proteins in ovariectomized hypertensive rats [6]. Our previous studies indicate that Fas receptor-dependent and mitochondrial-dependent apoptotic pathways were activated in hypertension [22] or ovariectomized [16,23] rats’ hearts. This study was undertaken to understand whether cardiac abnormalities in hypertension with coexisting ovariectomy are associated with more activated Fas-dependent and mitochondrial-dependent apoptotic pathways. We hypothesized that the coexistence of hypertension and ovariectomy might attenuate the estrogen receptor-related survival pathway, as well as might be predisposed to a more activated cardiac Fas receptor and mitochondrial-mediated cardiac apoptotic pathways.

## 2. Results

### 2.1. Body Weight and Cardiac Characteristics

The Wistar Kyoto (WKY) rat and the spontaneously hypertensive (SHR) rat inbred strains are well-established models for human hypertension. Therefore, we observed that systolic blood pressure (SBP), diastolic blood pressure (DBP) and mean arterial pressure (MAP) were significantly elevated in the SHR-Sham group when compared with the WKY group, but did not further influence the SHR with ovariectomized (SHR-OVX) group when compared with the SHR-Sham group (Table 1). The SHR-OVX group weighed about 16% more than the age-matched rats regardless of the SHR-Sham and WKY groups (Table 1). The uterine weight of the SHR-OVX group was significantly decreased relative to the SHR-Sham and WKY groups (Table 1). The whole heart weight (WHW) and whole heart weight/tibia length (WHW/TL) were increased in the SHR-Sham group when compared with the WKY group and further increased in the SHR-OVX group when compared with the SHR-Sham group (Table 1). The left ventricular weight (LVW) and left ventricular weight/tibia length (LVW/TL) were increased in the SHR-OVX when compared with the other two groups (Table 1).

### 2.2. Cardiac Histopathological Changes

**Figure 1:**
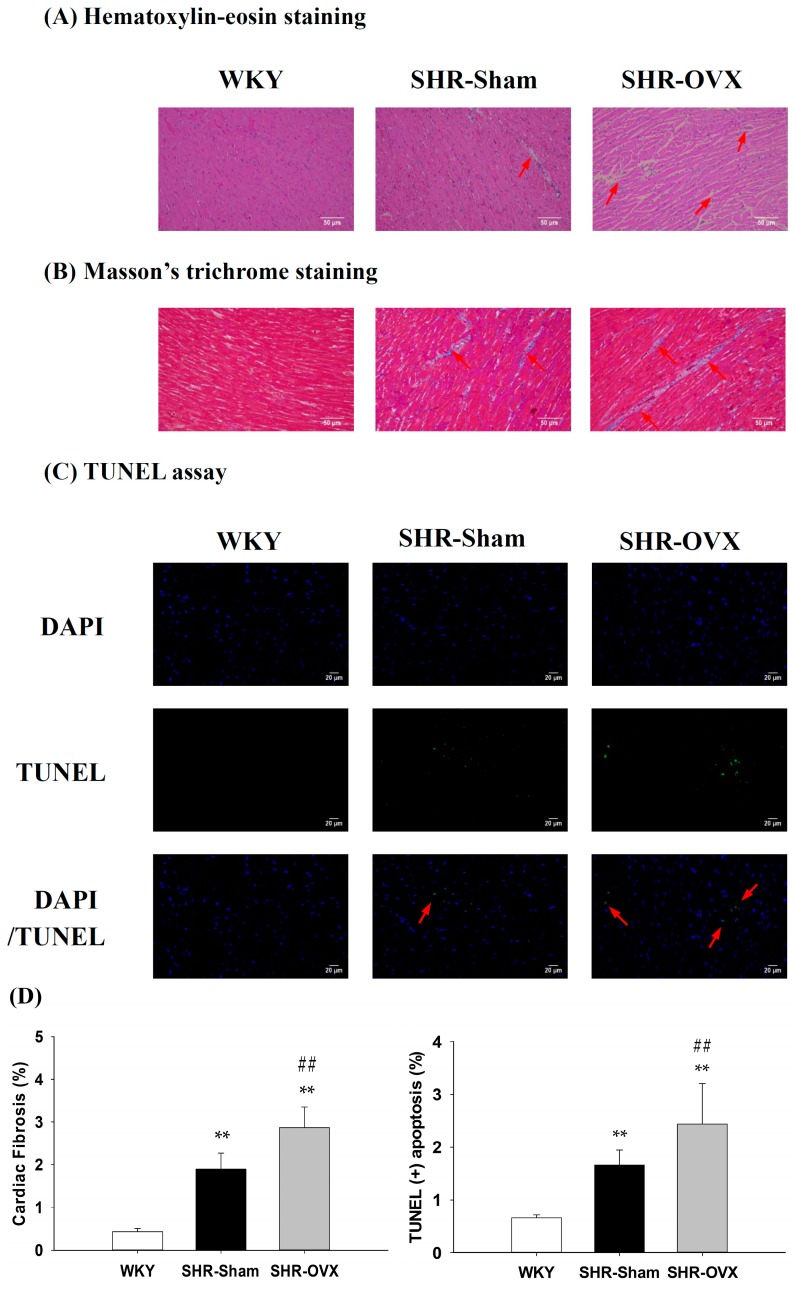
Representative histopathological analyses of cardiac sections from the left ventricles in the Wistar Kyoto rats (WKY), SHR with sham-operated (SHR-Sham) and SHR with bilateral ovariectomized (SHR-OVX) groups were performed with (**A**) hematoxylin-eosin staining (interstitial space: wide, arrows indicated) and (**B**) Masson’s trichrome staining (fibrosis: blue color, arrows indicated). The images of the myocardial architecture are magnified 200×; (**C**) Representative stained apoptotic cells of cardiac sections were performed by staining with 4,6-diamidino-2-phenylindole (DAPI) staining (**top**, blue spots) and the terminal deoxynucleotidyl transferase dUTP-mediated nick-end labeling (TUNEL) assay (**bottom**, green spots, arrows indicated) The images of myocardial architecture are magnified 400×; (**D**) The bar represents the percentage of the blue area to the field area in Masson’s trichrome staining, and the percentage of TUNEL apoptosis was expressed as the ratio of TUNEL-positive cells relative to total DAPI-stained nuclei and indicates mean values ± SD (*n* = 6 in each group); ** *p* < 0.01 denotes significant differences from the WKY group; ## *p* < 0.01 denotes the significant differences between the SHR-Sham group and the SHR-OVX group.

To investigate whether there were changes in cardiac architecture and fibrosis in the coexistence of hypertension and ovariectomy, we conducted a histopathological analysis of the left ventricular slices with H&E staining and Masson’s trichrome staining on hearts from the WKY, SHR-Sham and SHR-OVX groups. We observed that the left ventricles of the SHR-Sham group showed abnormal myocardial architecture with increased cardiac interstitial space and fibrosis relative to the WKY group, and even greater increases were observed in the SHR-OVX group when compared with the SHR-Sham group (Figure 1A,B,D).

### 2.3. TUNEL-Positive Apoptotic Cells of Left Ventricle

To clarify the apoptotic activity in cardiac tissues in the coexistence of hypertension and ovariectomy, a TUNEL assay and DAPI staining were measured in the left ventricular slices from the WKY, SHR-Sham and SHR-OVX groups. We observed the left ventricles of the SHR-Sham group to have a greater number of TUNEL-positive cardiac cells than those in the WKY group, with a further increase in the SHR-OVX group when compared with the SHR-Sham group (Figure 1C,D).

### 2.4. Components of Cardiac Estrogen Receptors

**Figure 2:**
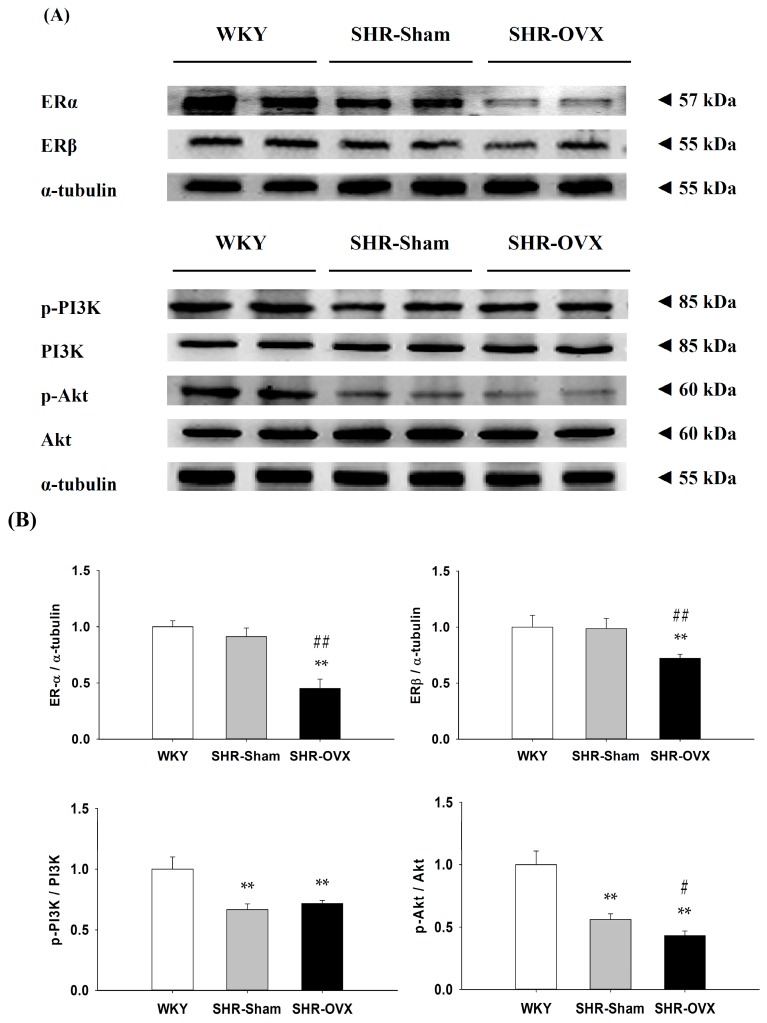
(**A**) The two representative protein products of estrogen receptor α (ERα) and estrogen receptor β (ERβ), p-PI3K, PI3K, p-Akt and Akt extracted from the left ventricles of excised hearts from the Wistar Kyoto rats (WKY), SHR with sham-operated (SHR-Sham) and SHR with bilateral ovariectomized (SHR-OVX) were measured by Western blot analysis; (**B**) The bars represent the relative protein quantification of ERα, ERβ, p-PI3K/PI3K and p-Akt/Akt on the basis of α-tubulin and the average protein expression levels in the WKY group normalized to one, and then, a relative protein expression was calculated for each condition. Data presented as the mean values ± SD (*n* = 8 in each group); ** *p* < 0.01 denotes significant differences from the WKY group; *# p* < 0.05, ## *p* < 0.01 denote significant differences from the SHR-OVX group.

To investigate the components of cardiac estrogen receptors in the coexistence of hypertension and ovariectomy, the protein levels of ERα, ERβ, p-PI3K, PI3K, p-Akt and Akt in the left ventricles were excised from the WKY, SHR-Sham and SHR-OVX groups and were measured by Western blotting. When compared with the WKY and SHR-Sham groups, we observed that the ovariectomy caused the protein levels of ERα and ERβ to become more significantly decreased in the SHR-OVX group (Figure 2). When compared with the WKY group, the protein levels of p-PI3K/PI3K and p-Akt/Akt were significantly decreased in the SHR-Sham group, with further decreases in the protein level of p-Akt/Akt, but there were no further decreases in the protein levels of the p-PI3K/PI3K, which were observed in the SHR-OVX group when compared with the SHR-Sham group (Figure 2).

### 2.5. Cardiac Bcl-2 Family Survival Pathway

**Figure 3:**
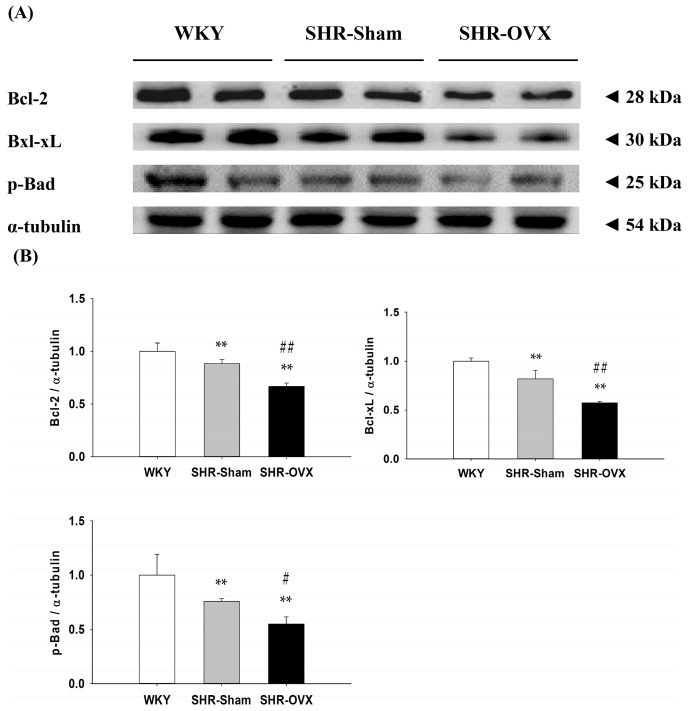
(**A**) The two representative protein products of Bcl-2, Bcl-xL and p-Bad extracted from the left ventricles of excised hearts from the Wistar Kyoto rats (WKY), SHR with sham-operated (SHR-Sham) and SHR with bilateral ovariectomized (SHR-OVX) were measured by Western blot analysis; (**B**) The bars represent the relative protein quantification of Bcl-2, Bcl-xL and p-Bad on the basis of α-tubulin and the average protein expression levels in the WKY group normalized to one, and then, a relative protein expression was calculated for each condition. Data presented as the mean values ± SD (*n* = 8 in each group); ** *p* < 0.01 denotes significant differences from the WKY group; # *p* < 0.05, ## *p* < 0.01 denote significant differences between the SHR-Sham group and the SHR-OVX group.

To investigate the components of the cardiac Bcl-2 family survival pathway in the coexistence of hypertension and ovariectomy, we measured the protein levels of Bcl-2, Bcl-xL and p-Bad in the left ventricles excised from the WKY, SHR-Sham and SHR-OVX groups by Western blotting. When compared with the WKY group, the protein levels of Bcl-2, Bcl-xL and p-Bad were significantly decreased in the SHR-Sham group, and even further decreases were observed in the SHR-OVX group when compared with the SHR-Sham group (Figure 3).

### 2.6. Cardiac Mitochondria-Dependent Apoptotic Pathways

**Figure 4:**
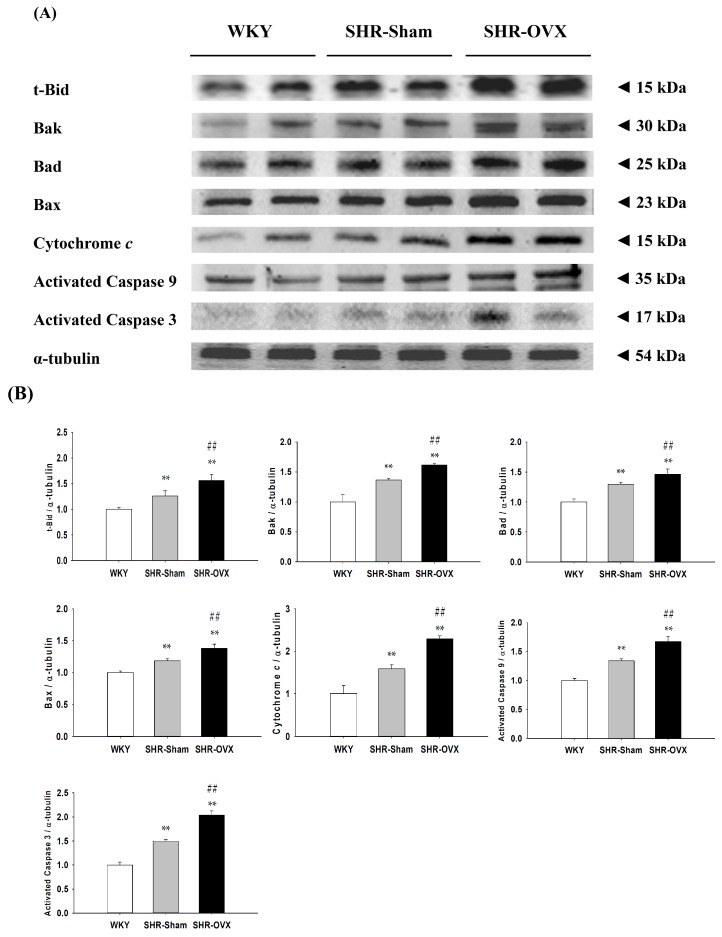
(**A**) The two representative protein products of t-Bid, Bak, Bad, Bax, cytochrome *c*, activated caspases 9 and activated caspases 3 extracted from the left ventricles of the excised hearts from the Wistar Kyoto rats (WKY), SHR with sham-operated (SHR-Sham) and SHR with bilateral ovariectomized (SHR-OVX) were measured by Western blot analysis; (**B**) The bars represent the relative protein quantification of t-Bid, Bak, Bad, Bax, cytochrome *c*, activated caspases 9 and activated caspases 3 on the basis of α-tubulin and the average protein expression levels in the WKY group normalized to one, and then, a relative protein expression was calculated for each condition. Data presented as the mean values ± SD (*n* = 8 in each group); ** *p* < 0.01 denotes significant differences from the WKY group; ## *p* < 0.01 denotes significant differences between the SHR-Sham group and the SHR-OVX group.

To further understand the cardiac mitochondria-dependent apoptotic signaling pathways in the coexistence of hypertension and ovariectomy, we measured the protein levels of t-Bid, Bak, Bad, Bax, cytochrome *c*, activated caspase 9 and activated caspase 3 in the left ventricles excised from the WKY, SHR-Sham and SHR-OVX groups by Western blotting. When compared with the WKY group, the protein levels of t-Bid, Bak, Bad, Bax, cytochrome *c*, activated caspase 9 and activated caspase 3 were significantly increased in the SHR-Sham group, and even further increases were observed in the SHR-OVX group when compared with the SHR-Sham group (Figure 4).

### 2.7. Cardiac Fas Receptor-Dependent Apoptotic Pathways

**Figure 5:**
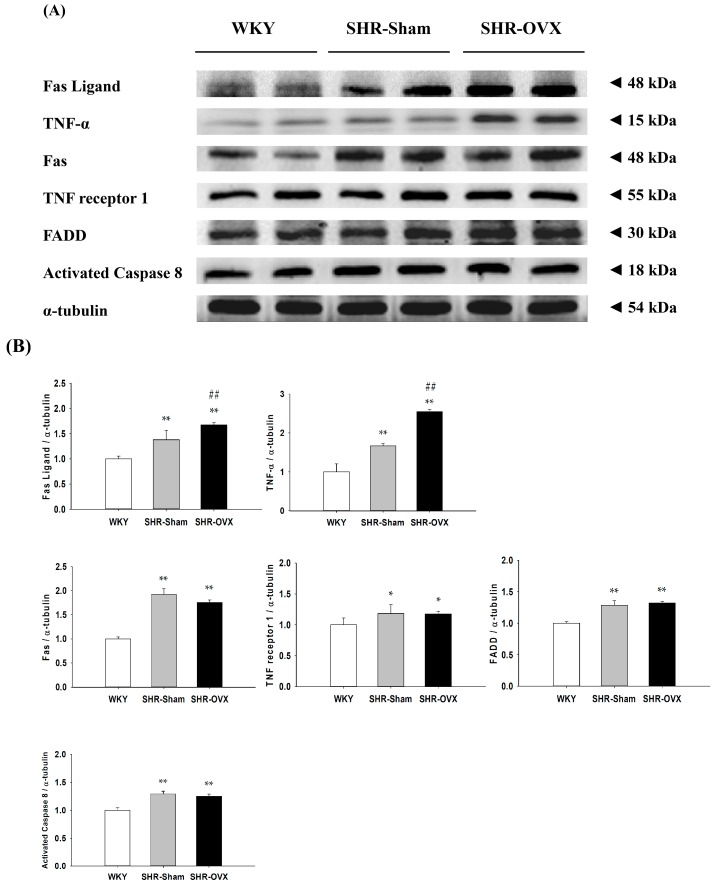
(**A**) The two representative protein products of Fas ligand, tumor necrosis factor-α (TNF-α), Fas death receptors (Fas), TNF receptor 1 (TNFR1), Fas-associated death domain (FADD) and activated caspases 8 extracted from the left ventricles of excised hearts from the Wistar Kyoto rats (WKY), SHR with sham-operated (SHR-Sham) and SHR with bilateral ovariectomized (SHR-OVX) were measured by Western blot analysis; (**B**) The bars represent the relative protein quantification of Fas ligand, tumor necrosis factor-α (TNF-α), Fas death receptors (Fas), TNF receptor 1 (TNFR1), Fas-associated death domain (FADD) and activated caspase-8 on the basis of α-tubulin and the average protein expression levels in the WKY group normalized to one, and then, a relative protein expression was calculated for each condition. Data presented as the mean values ± SD (*n* = 8 in each group); * *p* < 0.05, ** *p* < 0.01 denote significant differences from the WKY group; ## *p* < 0.01 denote significant differences between the SHR-Sham group and the SHR-OVX group.

To investigate the cardiac Fas receptor-dependent apoptotic signaling pathways in the coexistence of hypertension and ovariectomy, the protein levels of Fas ligand, tumor necrosis factor-α (TNF-α), Fas receptors (Fas), TNF receptor 1 (TNFR1), Fas-associated death domain (FADD) and activated caspase-8 in the left ventricles were excised from the WKY, SHR-Sham and SHR-OVX groups and were measured by Western blotting. When compared with the WKY group, the protein levels of Fas ligand, TNF-α, Fas, FADD and activated caspase-8 were significantly increased in the SHR-Sham group, with further increases in the protein levels of Fas ligand and TNF-α, but there were no further increases in the protein levels of the Fas receptors, TNFR1, FADD and activated caspase-8, which were observed in the SHR-OVX group when compared with the SHR-Sham group (Figure 5).

## 3. Discussion

**Figure 6:**
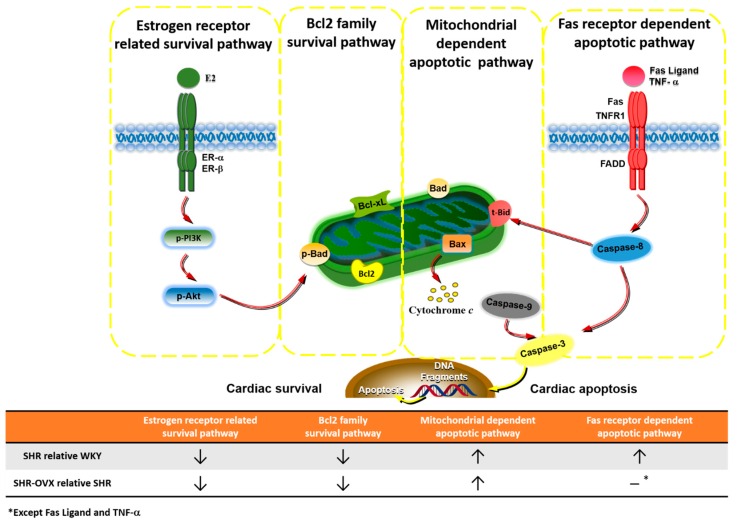
Proposed hypothesized diagram. Our hypothesis proposed that ovariectomy attenuated the estrogen receptor survival pathway (ERα, ERβ and p-Akt/Akt, as well as Bcl-2, Bcl-xL and p-Bad) and activated the cardiac mitochondria-dependent apoptotic pathway (t-Bid, Bak, Bad, Bax, cytochrome *c*, activated caspase-9 and activated caspase-3), but did not activate the cardiac Fas receptor-dependent apoptotic pathway (Fas ligand, TNF-α, Fas, TNFR1, FADD and activated caspase-8) in the coexistence of hypertension and ovariectomy.

Our main findings can be summarized as follows: (1) Elevated blood pressure was observed in hypertensive rats, but did not became more obvious when hypertension and ovariectomy coexisted; (2) Abnormal myocardial architecture enlarged the interstitial space and increased cardiac fibrosis, and more cardiac TUNEL-positive apoptotic cells were observed in hypertension. These increases became more obvious when hypertension and ovariectomy coexisted; (3) The cardiac estrogen receptor-related and Bcl-2 family pro-survival pathways decreased in hypertension and further decreased in hypertensive rats with the coexistent ovariectomy, which is based on a decrease in ERα, ERβ, p-Akt/Akt, Bcl-2, Bcl-xL and p-Bad; (4) The activity of the cardiac mitochondrial-dependent apoptotic pathway increased in the hypertension and further increased in hypertension with coexistent ovariectomy, evidence for which is based on increases in the t-Bid, Bak, Bad, Bax, cytochrome *c*, activated caspase-9 and activated caspase-3; (5) The activity of the cardiac Fas receptor-dependent apoptotic pathway increased in the hypertension, the evidence for which is based on increases in Fas ligand, TNF-α, Fas death receptors, TNFR1, FADD and activated caspase-8, but we did not observe further increases in hypertension with coexistent ovariectomy, except Fas ligand and TNF-α. Our expectation hypothesis was to examine cardiac abnormality in the coexistence of hypertension and ovariectomy that might be attenuated with the estrogen receptor-related survival pathway, as well as might be predisposed to more active cardiac Fas receptors and mitochondrial-mediated cardiac apoptotic pathways. However, our results from the present study did not fully support our expected hypothesis. We modified the hypothesis and proposed that cardiac abnormality in the coexistence of hypertension and ovariectomy might have attenuated the estrogen receptor-related survival pathway and might be predisposed to be more activated and cardiac mitochondria-dependent, but not the Fas receptor-dependent apoptotic pathway in the coexistence of hypertension and ovariectomy (Figure 6).

Menopause-related estrogen-deficiency involves the development of cardiovascular disease, especially age-related cardiac dysfunction and cardiac remodeling [7,23,24]. Hypertension causes pathological cardiac hypertrophy and is recognized as the most important predictor of cardiovascular morbidity and mortality, as well as an important risk factor for heart failure [25]. Either hypertension or ovariectomy has been known as a high risk for myocardial damage with increased activities of collagenases, which contribute to the development of cardiac fibrosis and remodeling [26,27]. In the present study, we observed that cardiac hypertrophy, enlarged interstitial space, myocardial disarray and cardiac fibrosis appear to have increased in hypertensive rats and appear to be more severe in the simultaneous presence of both hypertension and ovariectomy. Additionally, many lines of evidence have suggested that excess activity of the sympathetic nervous system may increases blood pressure [28]. We observed that SBP, DBP and MAP were increased in hypertensive rats. This suggested that the adrenergic system, such as α/β-adrenergic imbalance [29,30] and α/β-adrenergic receptor expression [31], may play an important role in the pathogenesis of hypertension and heart failure [29,30]. However, we observed that ovariectomy did not further affect blood pressure levels in female hypertensive rats. Our findings are consistent with those of some studies that showed that hypertension was not exacerbated in hypertensive rats after ovariectomy [32,33]. Indeed, menopause is characterized by increases in blood pressure [1]. However, the blood pressure does not increase during the menopause transition, but rather, the increase in blood pressure after menopause takes an average of five to 20 years to develop. This suggested that the deficit of female hormones may not be the only contributing factor for the elevated blood pressure [34,35]. In contrast, other studies showed that ovariectomies accelerated hypertension development in the aging female Dahl salt-sensitive models [36]. The influence of an ovariectomy on hypertension development remains. The reasons for these discrepancies are unclear, but may involve strain difference factors, such as sodium intake, estrogen, age and diet. In sum, it is conceivable that estrogen deficiency could be a possible factor, which might cause altered cardiac ventricular structure and function in hypertension with coexistent ovariectomy, rather than hypertension dominance.

In the present study, we found that the cardiac ERα and ERβ protein levels significantly downregulated and also reduced the activation of PI3K/Akt pathways in ovariectomized hypertension. Our results are similar to the previous report that demonstrated that ablation of the ERβ gene significantly decreased myocardial PI3K/Akt activation, which was associated with increased cardiac apoptotic expression in females after ischemia/reperfusion injury [37]. This implies possibly that ovariectomy through downregulation of the estrogen receptor-mediated PI3K/Akt and anti-apoptotic signaling pathway leads to cardiac apoptosis. Our findings suggest that cardiac abnormalities in hypertension with a coexistent ovariectomy is associated with downregulation of the estrogen receptor-mediated PI3K/Akt and Bcl-2 family survival signaling pathways.

Apoptosis occurring in terminally-differentiated cardiomyocytes is a critical pathological mechanism leading to heart failure [21]. Therefore, we set out to investigate whether an understanding of the process of apoptosis could allow for the development of novel strategies to reverse or attenuate heart failure. In the present study, we observed that the cardiac Fas receptor and mitochondria-dependent apoptotic pathways were activated in hypertension, which are consistent with our previous studies [22]. Previous studies reported that cardiomyocytes apoptosis is mediated by Bcl-2 and Bax genes and proteins in ovariectomized hypertensive rats [6]. The current report presents that the coexistence of hypertension and ovariectomy might predispose to a more activate cardiac mitochondria-dependent pathway, but no further changes in the Fas receptor-dependent apoptotic pathway. Our findings strongly suggest that cardiac apoptosis induced by the coexistence of hypertension and ovariectomy is related to the mitochondria-dependent apoptotic pathway. Additionally, we observed TNF-α significantly upregulated in hypertension alone and the coexistence of hypertension and ovariectomy. A previous study has indicated inflammation to be a key in the pathogenesis of hypertension and cardiovascular disease [38]. TNF-α is a pro-apoptotic molecule and pro-inflammatory factor [39]. Early studies demonstrated that TNF-α may be one of several potentially important maladaptive mechanisms involved in a maladaptive response leading to heart failure [40]. One study also reported that OVX induced an increase in genes mediating inflammation, such as IL-6 and TNF-α [41]. This implies possibly that the coexistence and independence of ovariectomy and hypertension may be associated with increased cardiac inflammation. However, the mechanism of estrogen deficiency, TNF-α-related inflammatory production in hypertension with coexistent ovariectomy was not determined in this work. In addition, there are some potential confounding factors in the current study, such as weight gain, that can be observed after an ovariectomy in the current study. Previous studies have indicated that women became overweight or obese after menopause and, consequently, many health risks are increased [42]. Obesity or weight gain may lead to cardiac apoptosis [43,44,45,46], but it is still unclear whether the increased cardiac apoptotic activity is partially due to the deleterious factor of weight gain after an ovariectomy. Moreover, the coexistence and independence of ovariectomy and hypertension share many of the same or different disease processes and risk factors, such as weight change [42], increased inflammation [47], oxidative stress [48,49], renin-angiotensin system activity [6,26] and heart and renal changes [50]. This implies that other factors involved in these changes and further studies are required to evaluate these issues. Therefore, we need to make a cautious note that any detrimental effect of hypertension with coexistent ovariectomy on hearts cannot be isolated to one specific factor, such as body weight changes, the estrogen receptor mechanism, dyslipidemia, lipid accumulation, insulin resistance, oxidative stress, the renin-angiotensin system, inflammation or unclear interacting factors. Taken together, this study provides that the coexistence of hypertension and ovariectomy additively increases cardiac apoptosis, which might help to explain the pathophysiology of heart failure or hypertension-associated heart diseases in severely hypertensive women with postmenopausal or early oophorectomy.

### Hypothesized and Clinical Application

Hypertension and postmenopause are considered to be major risk factors in the development of heart failure. Because cardiac tissues are difficult to be sampled from menopausal humans’ hearts, the current hypertensive undergoing a bilateral ovariectomy animal model should provide an important mechanism for explaining cardiac diseases in the simultaneous postmenopausal and hypertensive status. Our current findings revealed that the coexistence of hypertension and ovariectomy attenuated the estrogen receptor survival pathway and appeared to additively increase the cardiac mitochondria-dependent, but not the Fas receptor-dependent apoptosis pathway, which might provide one possible mechanism behind the development of heart failure in the simultaneous postmenopausal and hypertensive status. This study raises further questions, as to whether anti-apoptotic therapy might be beneficial to attenuate cardiac apoptosis, such as exercise training [22,51]. Of course, further therapeutic or clinical studies are required to clarify the effects of treatments or possible mechanisms in postmenopausal women with coexistent hypertension-related heart abnormalities.

## 4. Experimental Section

### 4.1. Animals

Twenty-eight female spontaneously hypertensive rats (SHR) and fourteen age-match female Wistar Kyoto rats (WKY) were purchased from the National Laboratory Animal Breeding and Research Center, Taipei, Taiwan. The rats were housed in a temperature-controlled room of 25 ± 1 °C with a 12-h dark/light cycle and fed with standard laboratory chow (Lab Diet 5001; PMI Nutrition International, Brentwood, MO, USA) and water ad libitum. All protocols were handled according to the Institutional Animal Care and Use Committee of the University of Taipei Animal Center (IACUC Approval No: 20110003, date: 28 December 2011), and the principles of laboratory animal care (NIH publication) were followed.

### 4.2. Ovariectomized Rat Model

The ovariectomy was performed following the technique described by Lee [23]. At fifteen-weeks-old, SHR rats were randomly assigned to the sham-operated rats (SHR-Sham, *n* = 14) and bilateral ovariectomized rats (SHR-OVX, *n* = 14). All rats were operated on with survival surgical procedures with an aseptic technique. The OVX rats were anesthetized with an injection of sodium pentobarbital (40 mg/kg; Intraperitoneal), and the bilateral ovaries were removed. The sham-operated rats underwent the same surgical procedure except for the removal of the ovaries. After surgery, each rat was injected with penicillin-G procaine (0.2 mL, 20,000 IU; Intramuscular) and allowed to recover for 1 week. All rats were anaesthetized with 2% isoflurane delivered in oxygen (95% O_2_ and 5% CO_2_), and all efforts were made to minimize suffering.

### 4.3. Resting Blood Pressure and Heart Rate

The resting blood pressure and heart rate were measured by a noninvasive tail-cuff pressure meter system (LE5001, Panlab, Wood Dale, IL, USA); the systolic/diastolic blood pressure (SBP/DBP), mean arterial pressure (MAP) and heart rate (HR) were measured in conscious rats. For accurate measurement, the average of five consecutive readings was used.

### 4.4. Cardiac Characteristics

The hearts of rats in the three groups were excised at twenty five weeks old and cleaned with phosphate-buffered saline (PBS, pH 7.4). The left ventricles were separated from whole hearts. The right tibias were separated, and right tibia lengths (TL) were measured. The whole heart weight (WHW) and the left ventricle weight (LVW) were measured. The LVW/WHW, WHW/BW, LVW/BW, WHW/TL and LVW/TL were calculated.

### 4.5. Hematoxylin-Eosin Staining

The hearts of six rats from each group (from a total of fourteen rats per group) were soaked in 4% formalin, dehydrated through graded alcohols and embedded in paraffin wax. Paraffin sections 2 μm thick were cut from paraffin-embedded tissue blocks. The tissue sections were de-paraffinized, hydrated, boiled in Trilogy solution (Cell Marque, Rocklin, CA, USA) for 20 min and rinsed in PBS (pH 7.2). Next, all slices were dyed with hematoxylin (Merck, Darmstadt, Germany) for 5 min and then washed in running tap water for 5 min. All slides were then immersed in eosin (Merck) for 5 min followed by washing in running tap water for 5 min. Each slide was dehydrated through graded alcohols (75%, 85%, 90% and 100%). Finally, they were soaked in xylene twice. Photomicrographs were obtained using a phase-contrast microscope (200×, Olympus BX43, Tokyo, Japan).

### 4.6. Masson’s Trichrome Staining

The hearts of six rats from each group (from a total of fourteen rats per group) were soaked in 4% formalin, dehydrated through graded alcohols and embedded in paraffin wax. Paraffin sections 2 μm thick were cut from the paraffin-embedded tissue blocks. The tissue sections were de-paraffinized, hydrated, boiled in Trilogy solution (Cell Marque, Rocklin, CA, USA) for 20 min and rinsed in PBS. Next, all slices were dyed with Masson’s trichrome kit (Scytek Laboratories, Logan, UT, USA). In brief, the sections were soaked in a warmed Bouin’s solution at 60 °C for 45 min, then washed in running tap water until the yellow color in the samples disappeared. All slides were immersed in Weigert’s hematoxylin for 5 min followed by washing in running tap water for 2 min. The slides were stained with acid fuchsine for 15 min and rinsed in distilled water. Next, the slides were treated with phosphomolybdic acid solution for 10 min and then immediately stained with methyl blue solution for 10 min. Slides were rinsed with distilled water and treated with 1% acetic acid solution for 3 min. Each slide was dehydrated through 2 changes of alcohol (95%, 95%, 100% and 100%). Finally, they were soaked in xylene twice. Photomicrographs were obtained using a phase-contrast microscope (200×, Olympus BX43, Tokyo, Japan). The quantification of fibrotic areas (stained blue) and myocardial areas (stained red) was performed using ImageJ analysis software. The fibrosis percentage of the left ventricular area was obtained by calculating the ratio of fibrotic area to the myocardial area.

### 4.7. DAPI and TUNEL Staining

For the terminal deoxynucleotidyl transferase dUTP-mediated nick-end labeling (TUNEL) assay, the sections were incubated with proteinase K for 30 min, washed in phosphate-buffered saline (PBS) two times for 5 min, followed by incubation with permeabilization solution (0.1% Triton X-100 in 0.1% Sodium Citrate, freshly prepared) for 8 min and then washed in PBS two times for 5 min. The sections were soaked in a blocking buffer (Tris-HCl, 0.1 M, pH 7.5 containing 3% bovine serum albumin (BSA) and 20% normal bovine serum) for 60 min and then washed in PBS two times for 5 min. The slides were incubated with terminal deoxynucleotidyl transferase (TdT) and fluorescein isothiocyanate-dUTP conjugated from the In Situ Cell Death Detection Kit, Fluorescein (Roche Applied Science) for 60 min and then washed in PBS two times for 5 min. The slides were mounted using DAPI-fluoromount G (Southern Biotech, Birmingham, AL, USA). TUNEL-positive nuclei (fragmented DNA) were fluoresced by bright green light at 450 to 500 nm. The nucleus position was fluoresced by blue light at 340 to 380 nm. Photomicrographs were obtained using a phase-contrast microscope (400×, Olympus BX43, Tokyo, Japan). The numbers of TUNEL-positive and DAPI-stained nuclei were determined, and the percentage of TUNEL-apoptosis was calculated as the ratio of TUNEL-positive relative to DAPI-stained nuclei from the left ventricular area.

### 4.8. Western Immunoblotting

The hearts of eight rats from each group (from a total of fourteen rats per group) were excised, cleaned and immediately frozen. The left ventricular tissues were homogenized in tissue protein extraction reagent (T-PER, Thermo Scientific, Rockford, IL, USA) containing phosphatase and a protease inhibitor cocktail (Roche Applied Science, Mannheim, Germany) at 4 °C. The supernatants were collected after centrifugation at 12,000× *g* for 40 min at 4 °C. The protein concentration was determined by the Bradford method (Bio-Rad Laboratories, Hercules, CA, USA). Protein samples (40 μg/lane) were separated on a 10% or 12% SDS polyacrylamide gel by electrophoresis (SDS-PAGE) with a constant voltage of 75 V. Electrophoresed proteins were transferred to a polyvinylidene difluoride (PVDF) membrane (0.45-μm pore size, Millipore, Bedford, MA, USA) with a transfer apparatus (Bio-red, Hercules, CA, USA). PVDF membranes were incubated in 5% non-fat dried milk in TBST buffer (25 mM Tris-HCl, 150 mM NaCl, pH 7.6 and 0.1% Tween-20). Primary antibodies including ERα, ERβ (Abcam, Cambridge, UK), p-PI3K, PI3K, p-Akt, Akt, Bcl-2, Bcl-xL, p-Bad, t-Bid, Bak, Bad, Bax, cytochrome *c*, caspase-9, caspase-3, Fas ligand, TNF-α, Fas receptors, TNF receptor 1, FADD, caspase-8 and α-tubulin (Santa Cruz Biotechnology, Santa Cruz, CA, USA) were diluted to 1:500 in antibody binding buffer overnight at 4 °C. The immunoblots were washed in a TBST buffer three times for 10 min and followed by incubation with an HRP-conjugated second antibody solution (1:5000 dilution, Santa Cruz Biotechnology) for 1 h at room temperature. The immunoblots were then washed in the TBST buffer three times for 10 min. The immunoblotted proteins were visualized using an enhanced chemiluminescence ECL Western Blotting Luminal Reagent (Millipore Corporation, Billerica, MA, USA) and quantified using a Fujifilm LAS-3000 chemiluminescence detection system (Fuji, Tokyo, Japan).

### 4.9. Statistical Analysis

Data for the weight index, echocardiography index and protein levels were compared between the WKY, SHR-Sham and SHR-OVX groups using one-way ANOVA with preplanned contrast comparison with the control group. WKY and SHR-OVX served as the negative control and the positive control, respectively. *p* < 0.05 was considered to be significant.

## 5. Conclusions

Our current findings that the coexistence of hypertension and ovariectomy attenuated the estrogen receptor survival pathway and appeared to additively increase the cardiac mitochondria-dependent, but not the Fas receptor-dependent apoptosis pathway, which might provide one possible mechanism behind the development of heart failure in hypertensive postmenopausal women.

## Figures and Tables

**Table 1:** Cardiac characteristics of WKY, SHR-Sham and SHR-OVX groups.

	WKY	SHR-Sham	SHR-OVX
**Number of Animals**	8	8	8
Body weight (g)	218 ± 14	219 ± 8	253 ± 8 **^,##^
Uterine weight (g)	0.46 ± 0.10	0.41 ± 0.09	0.08 ± 0.01 **^,##^
Resting HR (bpm)	374 ± 20	458 ± 19 **	463 ± 16 **
**Heart Weight Index**			
WHW (g)	0.78 ± 0.04	0.83 ± 0.04 *	0.93 ± 0.06 **^,##^
LVW (g)	0.57 ± 0.05	0.61 ± 0.03	0.65 ± 0.06 **
LVW (g)/WHW (g)	0.73 ± 0.03	0.73 ± 0.04	0.71 ± 0.05
WHW (g)/BW (g) * 10^3^	3.57 ± 0.34	3.80 ± 0.18	3.65 ± 0.18
LVW (g)/BW (g) * 10^3^	2.61 ± 0.31	2.78 ± 0.18	2.58 ± 0.23
WHW (g)/TL (mm) * 10^3^	19.87 ± 1.15	21.34 ± 0.93 *	23.64 ± 1.63 **^,##^
LVW (g)/TL (mm) * 10^3^	14.53 ± 1.24	15.58 ± 0.88	16.73 ± 1.85 **
**Blood Pressure**			
SBP (mmHg)	121 ± 7	169 ± 7 **	174 ± 2 **
DBP (mmHg)	83 ± 12	129 ± 10 **	134 ± 6 **
MAP (mmHg)	96 ± 9	141 ± 8 **	145 ± 6 **

Values are the means ± SD. Three groups: Wistar Kyoto rats (WKY), SHR with sham-operated (SHR-Sham) and SHR with bilateral ovariectomized (SHR-OVX). HR, heart rate; WHW, whole heart weight; LVW, left ventricular weight; BW, body weight; TL, tibia length; SBP, systolic blood pressure; DBP, diastolic blood pressure; MAP, mean arterial pressure. * *p* < 0.05, ** *p* < 0.01 denote significant differences from the SHR-Sham group; ^##^ *p* < 0.01 denotes significant differences between the SHR-OVX group and the SHR-OVX group.
